# Supplementary material for: The limits of sequence-based biosecurity screening tools in the age of AI-assisted protein design
Source: Front Bioeng Biotechnol. 2026 Jul 13;14:1858951. doi: 10.3389/fbioe.2026.1858951 (PMC13402867; doi:10.3389/fbioe.2026.1858951)
Supplement: Supplementary file 1 [file Supplementaryfile1.docx]

Supplementary Materials for

The limits of sequence-based biosecurity screening tools in the age of AI-assisted protein design

Bruce J. Wittmann^1^*, Nicole E. Wheeler^2,3^†‡, Steven T. Murphy^4^†, Tom Mitchell^4^†, Brittany Rife Magalis^5^†, Bryan T. Gemler^2,6^†, Kevin Flyangolts^2,7^†, James Diggans^2,8^†, Adam Clore^2,9^†, Jacob Beal^2,4^†, Craig Bartling^2,6^†, Tessa Alexanian^2,10^†, Eric Horvitz^1^*

^1^Microsoft, Office of the Chief Scientific Officer; Redmond, WA 98052, USA.

^2^International Gene Synthesis Consortium.

^3^Institute of Microbiology and Infection, Department of Microbes, Infections and Microbiomes, School of Infection, Inflammation and Immunology, College of Medicine and Health, University of Birmingham; Birmingham, B15 2TT, United Kingdom.

^4^RTX BBN Technologies; Cambridge, MA 02138, USA.

^5^University of Louisville; Louisville, KY 40292, USA

^6^Battelle; Columbus, OH 43201, USA.

^7^Aclid; New York, NY 10018, USA.

^8^Twist Bioscience; South San Francisco, CA 94080, USA.

^9^Integrated DNA Technologies Inc.; Coralville, IA 52241, USA.

^10^The International Biosecurity and Biosafety Initiative for Science (IBBIS); Geneva, 1207, Switzerland.

*Corresponding Authors: bwittmann@microsoft.com and horvitz@microsoft.com

^†^These authors contributed equally

^‡^Work completed while at listed affiliations. Now at Advanced Research + Invention Agency (ARIA), UK

# Table of Contents

Supplementary Data 2

Supplementary Tables 3

Supplementary Figures 4

Supplementary Data

Data S1. Sequence-level classification statistics by provider, fragment length, and tool. Column headers are as follows:

1. Provider: ID of the provider.
2. Fragment Length: The length of the fragment in nucleotides.
3. TPR: True positive rate $\left( \frac{TP}{TP+FN} \right)$.
4. FPR: False positive rate $\left( \frac{FP}{TN+FP} \right)$.
5. MCC: Matthew’s correlation coefficient. See *Methods* for definition.
6. TN: Number of true negatives.
7. TP: Number of true positives.
8. FN: Number of false negatives.
9. FP: Number of false positives.
10. Total: Sum of TN, TP, FN, and FP.
11. Tool: The ID of the tool (“A” for tools used in Wittmann et al., 2025, “B” for tools updated to be more effective at screening fragments).

Data S2. All data for experimental fragments. Column headers are defined as follows:

1. Fragment Index: Unique index assigned to a fragment of a specific sequence.
2. Flag: Whether this fragment was flagged as hazardous by the provider in the “Provider” column.
3. Tool: The generative model used to build the original sequence.
4. Protein: The protein used as a template. This uses the same random indexing scheme to deidentify proteins as used in Wittmann et al., 2025.
5. Condition: The strategy used to generate the protein. See “Materials and Methods: Reformulation” in the supplementary materials of Wittmann et al., 2025 for a description of the different conditions.
6. Sequence Name: The name of the sequence. This directly corresponds to the sequence name used in Wittmann et al., 2025.
7. Fragment Length: The length of the fragment in nucleotides.
8. Provider: ID of the provider/tool.
9. Upgraded: Whether or not the row corresponds to the most up-to-date BSS from the provider. This will be “TRUE” for Providers 1 and 2, Tools A, as well as for Providers 3 and 4, Tools B. It will be “FALSE” for Providers 3 and 4, Tools A.
10. DNA Fragment: The DNA sequence of the fragment.
11. Protein Fragment: The amino acid sequence of the fragment translated using the reading frame of the original, full-length sequence.
12. DNA Fragment (WT): The DNA sequence of the analogous wild-type fragment.
13. Protein Fragment (WT): The amino acid sequence of the analogous wild-type fragment.
14. Protein Frac Identity: The fractional sequence identity between the sequences in the “Protein Fragment” and “Protein Fragment (WT)” columns.
15. DNA Frac Identity: The fractional sequence identity between the sequences in the “DNA Fragment” and “DNA Fragment (WT)” columns.
16. Protein LCS: The longest common substring shared between the sequences in the “Protein Fragment” and “Protein Fragment (WT)” columns.
17. DNA LCS: The longest common substring shared between the sequences in the “DNA Fragment” and “DNA Fragment (WT)” columns.

Data S3. All data for negative control fragments. Column header definitions match those used for Data S2, with the following minor change:

1. Protein: Rather than a random index identifier, this is either the PDB ID or the NCBI Accession Number of the protein.

Supplementary Tables

**Table S1. True positive rate and false positive rate by tool and fragment length.**

|  | **Provider 1** | | **Provider 2** | | **Provider 3** | | | | **Provider 4** | | | |
| --- | --- | --- | --- | --- | --- | --- | --- | --- | --- | --- | --- | --- |
|  | **Tool A** | | **Tool A** | | **Tool A** | | **Tool B** | | **Tool A** | | **Tool B** | |
| **Fragment Length (nt)** | **TPR** | **FPR** | **TPR** | **FPR** | **TPR** | **FPR** | **TPR** | **FPR** | **TPR** | **FPR** | **TPR** | **FPR** |
| **25** | 0.0716 | 0.0000 | 0.4300 | 0.0000 | 0.0000 | 0.0000 | 0.0000 | 0.0000 | 0.0000 | 0.0290 | 0.0000 | 0.0000 |
| **50** | 0.7435 | 0.0000 | 0.9221 | 0.0000 | 0.0000 | 0.0010 | 0.6748 | 0.0010 | 0.9935 | 0.9778 | 0.8812 | 0.1988 |
| **75** | 0.9301 | 0.0010 | 0.9450 | 0.0000 | 0.0107 | 0.0010 | 0.9602 | 0.0010 | 0.9853 | 0.9411 | 0.9019 | 0.1342 |
| **100** | 0.9613 | 0.0010 | 0.9595 | 0.0000 | 0.1789 | 0.0000 | 0.9809 | 0.0000 | 0.9767 | 0.8089 | 0.9061 | 0.1158 |
| **125** | 0.9715 | 0.0010 | 0.9704 | 0.0010 | 0.3678 | 0.0087 | 0.9853 | 0.0087 | 0.9728 | 0.7896 | 0.8970 | 0.1149 |
| **150** | 0.9737 | 0.0010 | 0.9729 | 0.0029 | 0.5473 | 0.0077 | 0.9866 | 0.0077 | 0.9609 | 0.7712 | 0.8839 | 0.1139 |
| **175** | 0.9804 | 0.0010 | 0.9680 | 0.0000 | 0.7519 | 0.0048 | 0.9867 | 0.0048 | 0.9393 | 0.7500 | 0.8630 | 0.1100 |
| **200** | 0.9860 | 0.0000 | 0.9594 | 0.0000 | 0.8489 | 0.0000 | 0.9771 | 0.0000 | 0.9299 | 0.7210 | 0.8609 | 0.1110 |

Table S2. Detection rate in prior work of full-length more-probably-functional synthetic homologs of SOCs used to build fragments (Wittmann et al., 2025).

| **Provider** | **Tool** | **Detection Rate** |
| --- | --- | --- |
| 1 | A | 0.9897 |
| 2 | A | 0.9766 |
| 3 | A | 0.9862 |
| 4 | A | 0.9378 |

Supplementary Figures


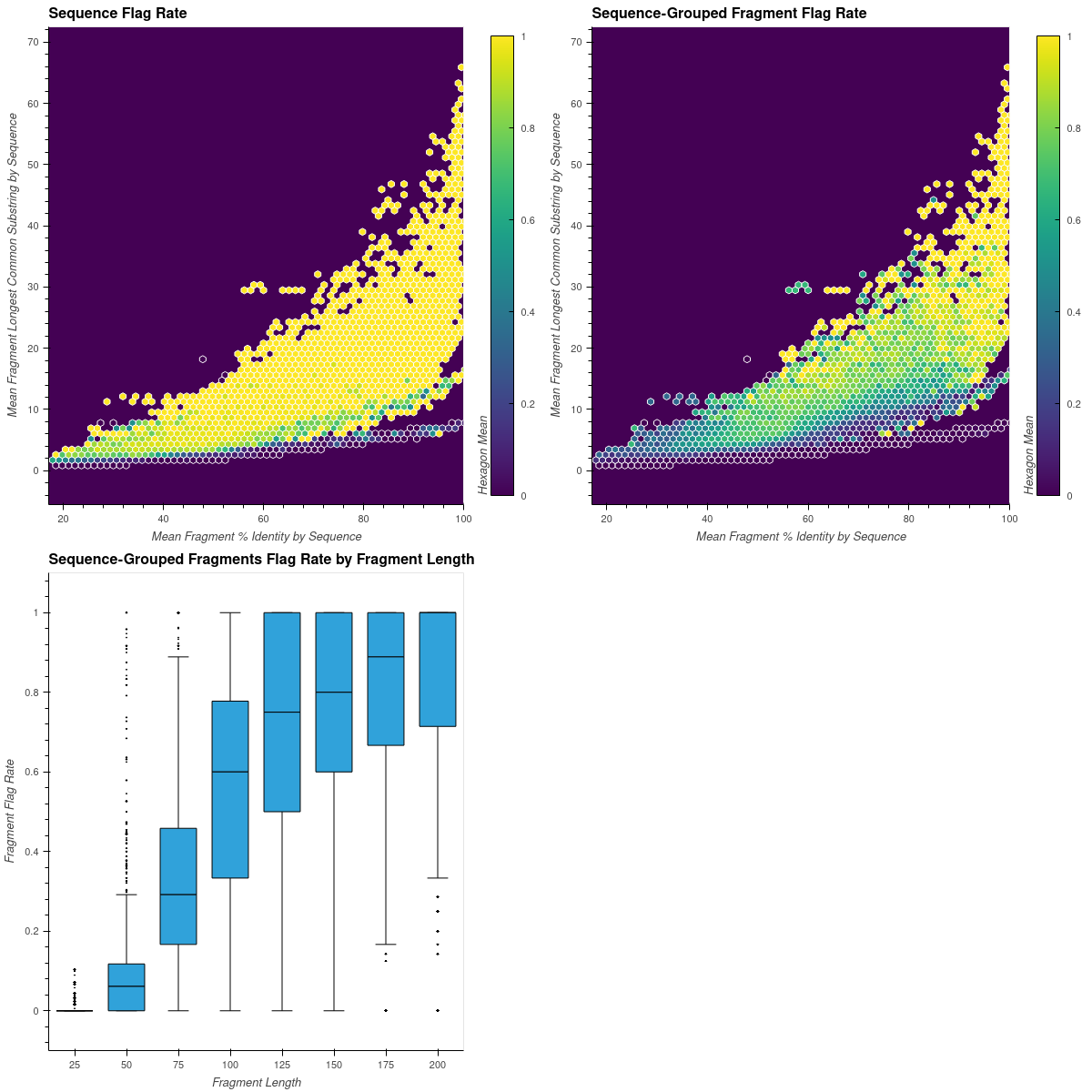


Fig. S1. Alternate representations of data displayed in Fig. 2 and Fig. 3 for Provider 1, Tool A. The first panel (top left) shows, for a given full-length sequence, the average fragment percent identity (x-axis) against the average longest common subsequence (y-axis). Each hexagon is colored according to the rate at which *at least one fragment* derived from the sequences it contains were flagged. That is, Panel 1 is a finer-grained representation of the data in Fig. 2. The x- and y-axes of the second panel (top right) have the same meaning as the first panel, only here each hexagon is colored according to the average rate of the frequency with which fragments derived from the sequences it contains were flagged. Put differently, Panel 2 is a coarser-grained representation of the data in Fig. 3 where the mean is taken over all fragments in a sequence before the binning operation is applied to color the hexagons. The final panel (bottom left) shows the same information as the second—the fraction of fragments flagged by sequence—only now plotted against the fragment length and displayed as a box-and-whisker plot rather than binning via hexagons.


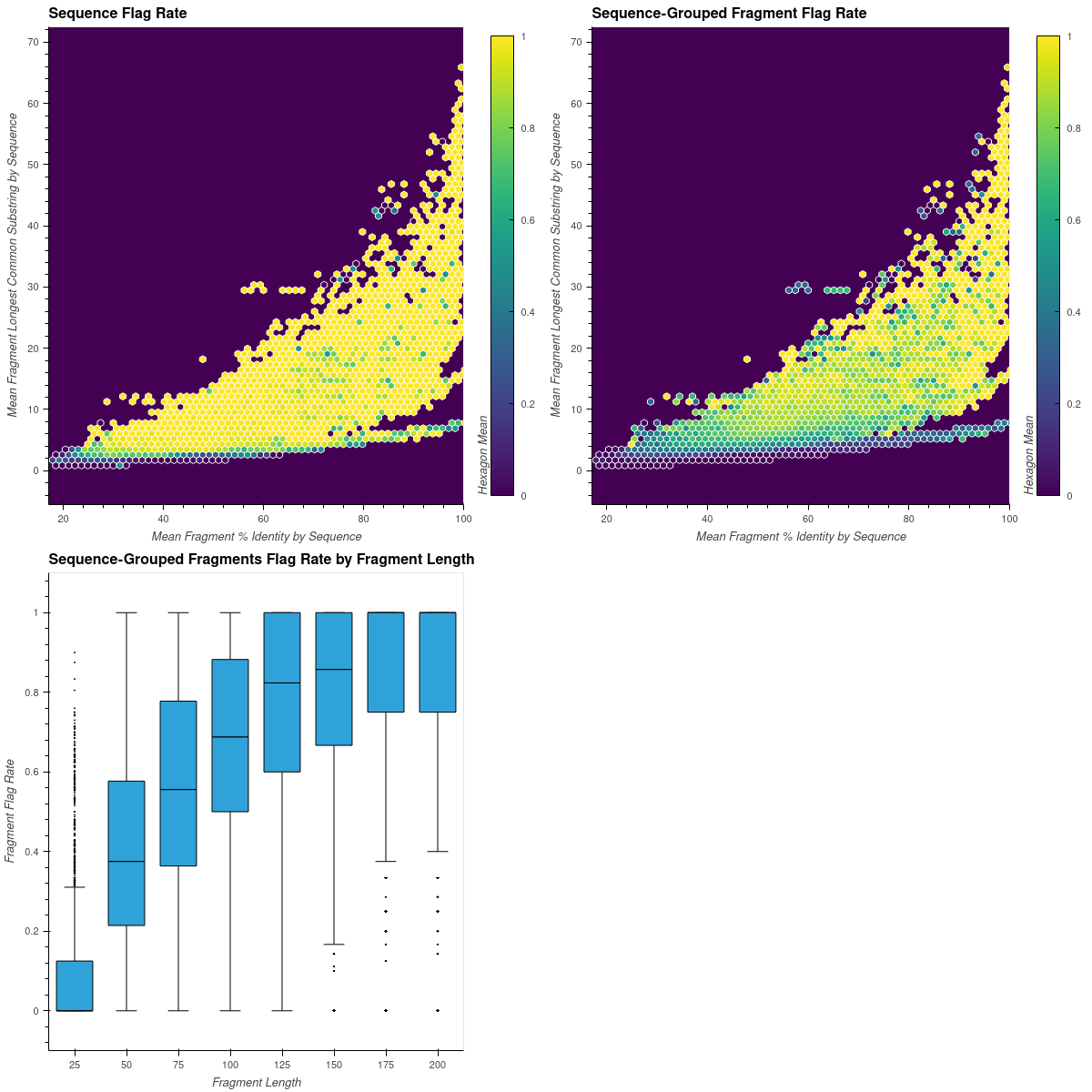


Fig. S2. Identical to Fig. S1, but for Provider 2, Tool A.


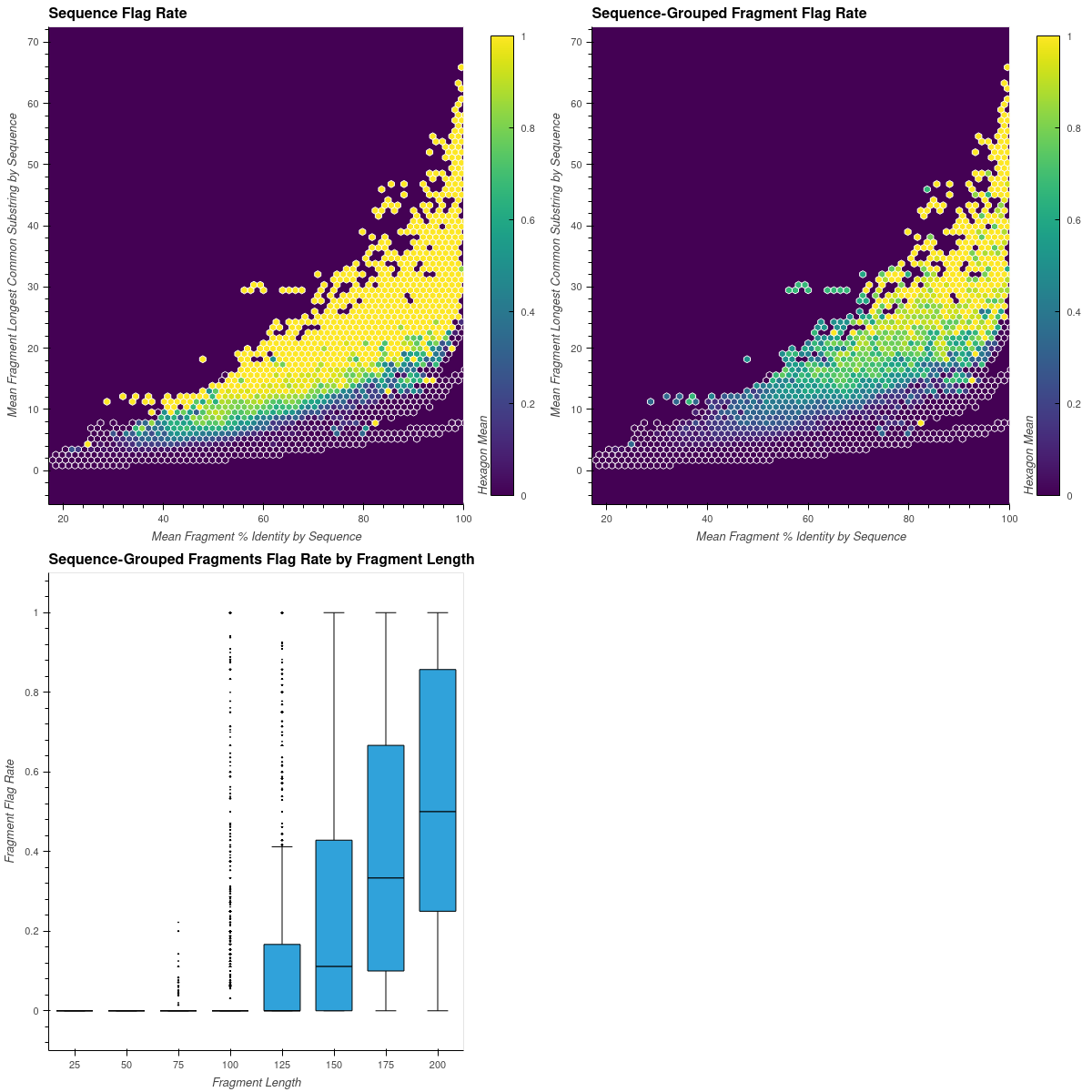


Fig. S3. Identical to Fig. S1, but for Provider 3, Tool A.


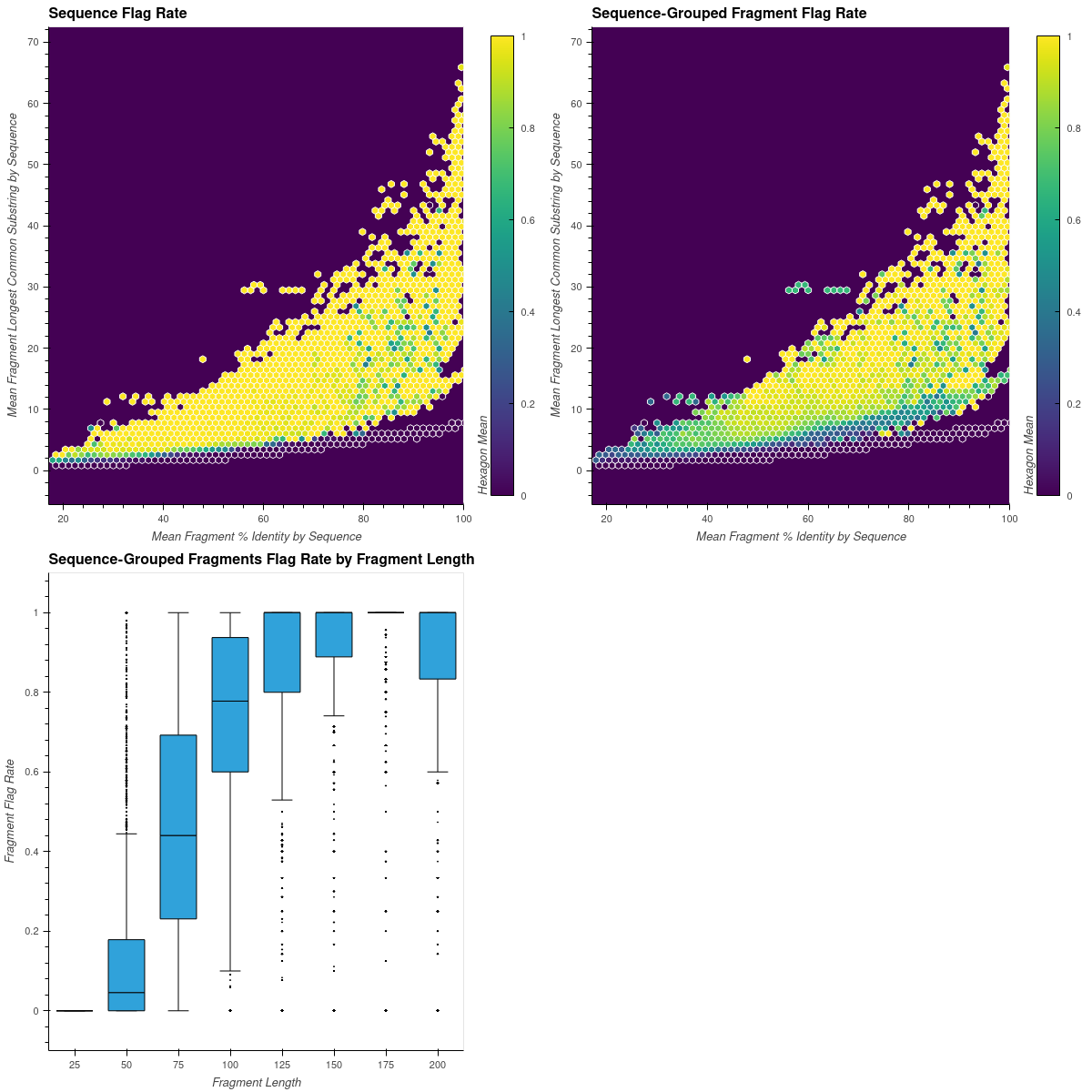


Fig. S4. Identical to Fig. S1, but for Provider 3, Tool B.


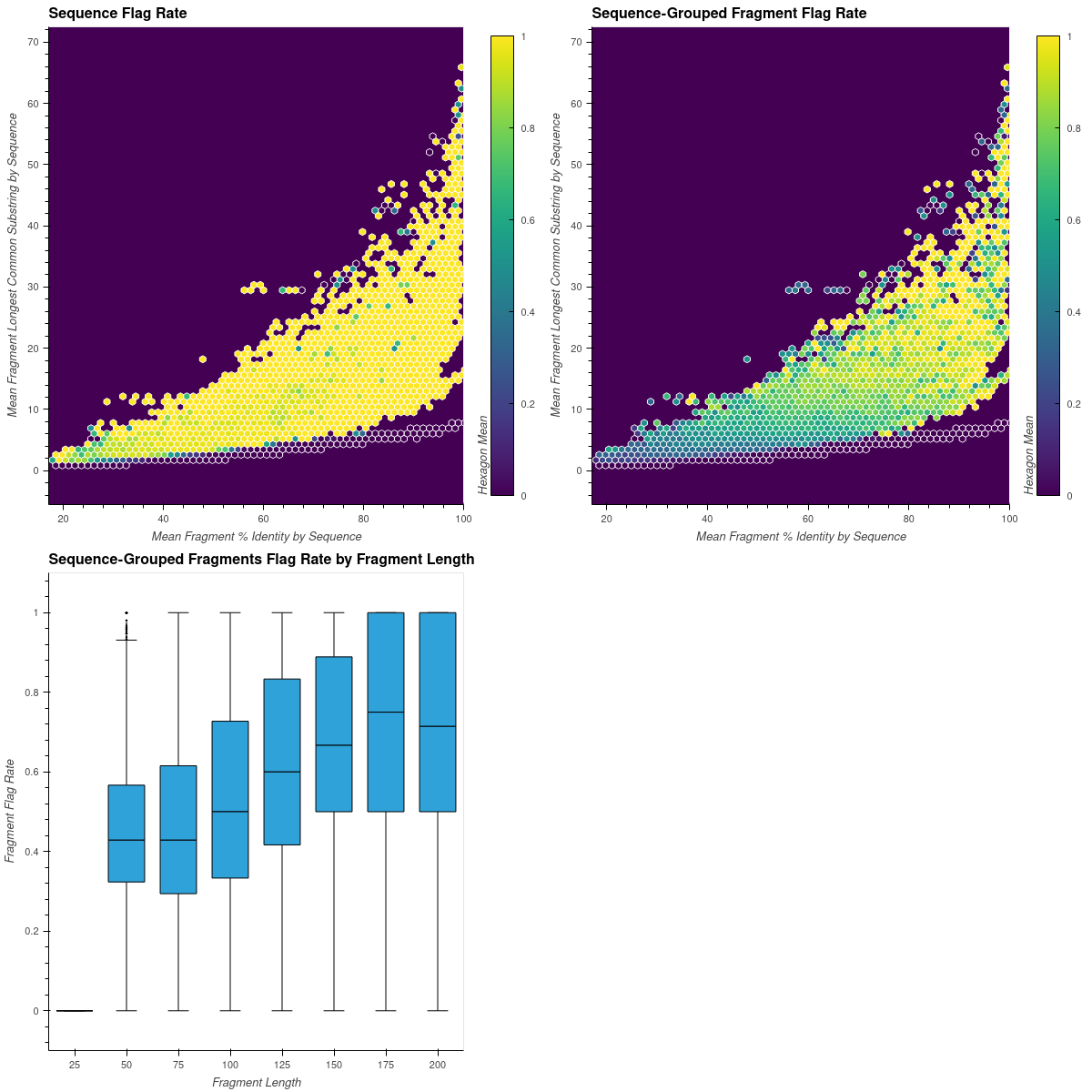


Fig. S5. Identical to Fig. S1, but for Provider 4, Tool A.


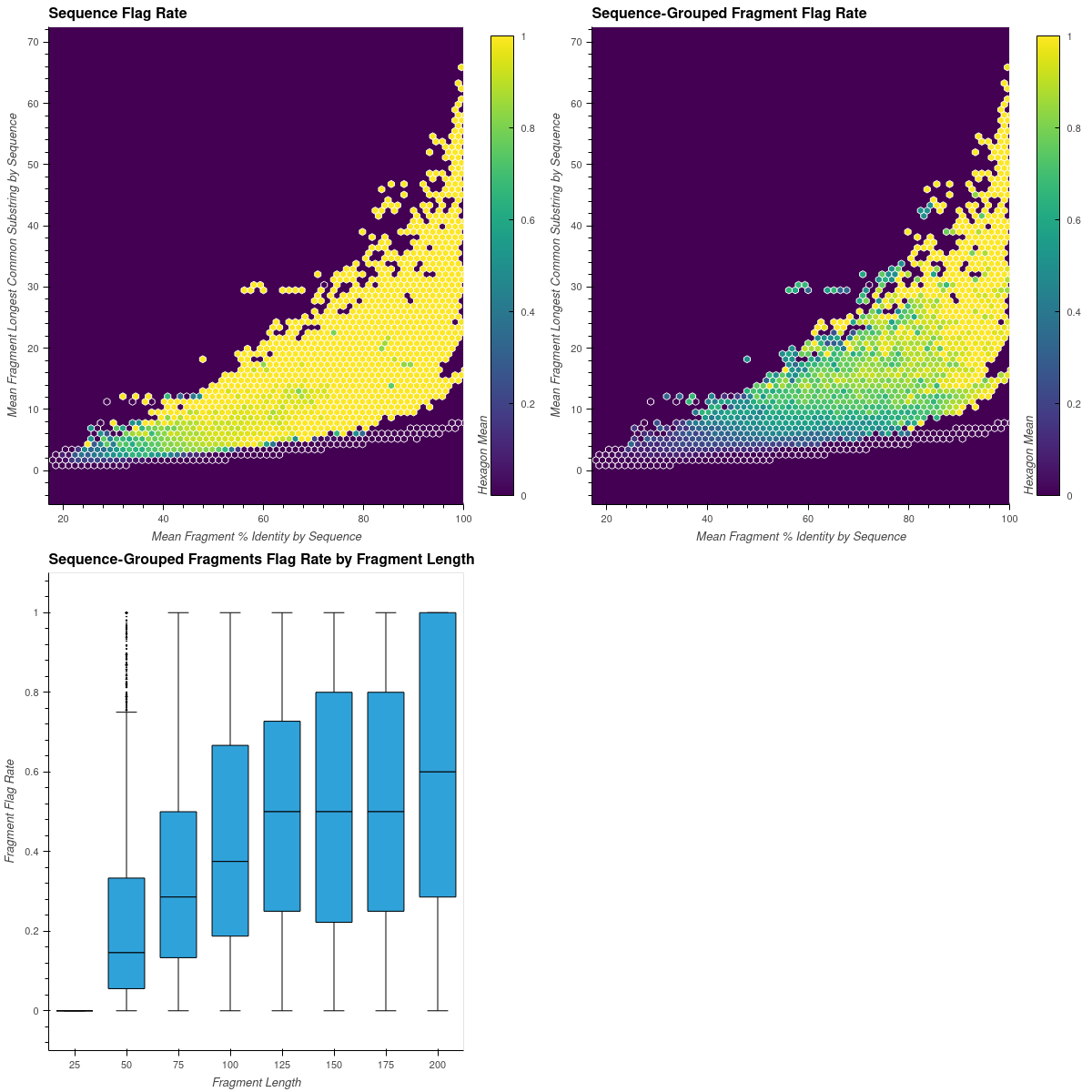


Fig. S6. Identical to Fig. S1, but for Provider 4, Tool B.
